# Renal function in Ethiopian HIV-positive adults on antiretroviral treatment with and without tenofovir

**DOI:** 10.1186/s12879-020-05308-9

**Published:** 2020-08-06

**Authors:** Daniel Yilma, Alemseged Abdissa, Pernille Kæstel, Markos Tesfaye, Mette F. Olsen, Tsinuel Girma, Christian Ritz, Henrik Friis, Åse B. Andersen, Ole Kirk

**Affiliations:** 1 Department of Internal Medicine, Jimma University, Jimma, Ethiopia; 2 Jimma University Clinical and Nutrition Research Centre, Jimma University, Jimma, Ethiopia; 3 Department of Nutrition, Exercise and Sports, University of Copenhagen, Copenhagen, Denmark; 4 Department of Laboratory Sciences and Pathology, Jimma University, Jimma, Ethiopia; 5 Department of Psychiatry, St. Paul’s Hospital Millennium Medical College, Addis Ababa, Ethiopia; 6 Department of Paediatrics and Child Health, Jimma University, Jimma, Ethiopia; 7 Department of Infectious Diseases, Rigshospitalet, Copenhagen, Denmark; 8 Research Unit of Infectious Diseases, Department of Clinical Research, University of Southern Denmark, Odense, Denmark

**Keywords:** HIV, Tenofovir, Renal function, Antiretroviral treatment

## Abstract

**Background:**

Limited data are available on the effect of antiretroviral treatment (ART) or Tenofovir disoproxil fumarate (TDF) on renal function in Ethiopians. We aimed to assess factors associated with renal function changes during the first year of ART with special focus on TDF.

**Methods:**

HIV positive persons who were ≥ 18 years of age and eligible for ART initiation were recruited. Creatinine measurement to estimate glomerular filtration rate (eGFR) and spot urine analyses were performed at baseline and after 3, 6 and 12 months of ART. Univariate and multivariate linear regression and univariate logistic regression were used to determine factors associated with eGFR as continuous and categorical variable respectively. A linear mixed model was used to assess 12 month eGFR difference in TDF and non-TDF based regimen.

**Result:**

Of 340 ART-naïve HIV patients with baseline renal function tests, 82.3% (279/339) were initiated on a TDF based ART regimen. All patients were on non-nucleoside reverse transcriptase inhibitors (NNRTI) based ART regimen. The median (IQR) change in eGFR with 12 months of ART was 0.8 (− 11.1; 10.0) ml/min/1.73m^2^. About 41 and 26.9% of HIV patients had a drop of greater than 3 and 10 mL/min/1.73 m^2^ in eGFR at 12 month, respectively. However, none of the HIV patients declined to < 60 ml/min/1.73m^2^ within 12 months. Moreover, none of the HIV patients had persistent proteinuria or glycosuria. Older HIV patients especially age > 45 years and those with unsuppressed viral load at 6 month of ART had a significantly lower eGFR at 12 months of ART initiation. However, there was no difference in 12 month eGFR between HIV patients initiated on TDF based regimen and non-TDF based regimen.

**Conclusion:**

Renal function remained stable with no difference between HIV patients treated with TDF or non-TDF NNRTI based ART regimen over 12 months. However, older HIV patients and those with unsuppressed viral load deserve special focus on renal monitoring. Data on long-term safety of TDF (> 1 year) is still warranted in this population.

## Background

Chronic kidney disease incidence has increased worldwide in HIV positive people [1], and renal dysfunction is associated with increased risk of death in HIV infection [2]. A relatively high prevalence of renal dysfunction was reported in several African countries in antiretroviral treatment (ART) naïve HIV positive people [3]. Low body mass index (BMI) [4, 5], low CD4 count [5], high viral load [6], concomitant hepatitis C virus (HCV) infection [6], and advanced stage of the disease [5, 7] were identified as risk factors for development of renal impairment in ART naïve HIV positive people in addition to the traditional risk factors for kidney diseases in the general population.

ART is the treatment for HIV associated nephropathies (HIVAN) [8] and early initiation of ART improves renal function [9]. However, long-term treatment with some antiretroviral drugs may result in renal impairment [10]. Tenofovir disoproxil fumarate (TDF), a nucleotide reverse transcriptase inhibitor, is a widely used antiretroviral drug and renal impairment [11, 12]. Fanconi syndrome [13], nephrogenic diabetes [14] and decline in estimated glomerular filtration rate (eGFR) [15, 16] have been reported with TDF use in HIV positive persons living in different settings. The World Health Organization (WHO) recommends to routinely determine estimated glomerular filtration rate (eGFR) before initiating and while on treatment on a TDF-containing regimen [17]. However, routine creatinine measurement is a challenge in sub-Saharan countries [18].

A number of studies conducted to look at the renal safety of TDF in sub-Saharan Africa and provides variable results. Most reported TDF use was associated with decline renal function [11] and some reported TDF had good renal safety [19, 20]. However, the larger cohort studies in Africa showed the association of TDF with decline renal function but the incidence of TDF induced nephrotoxicity is low [21–23].

Ethiopia introduced TDF as first line drug in national ART guideline in 2008 [24] but limited information is available on renal function change in HIV positive adults with ART initiation or TDF use. Moreover, most of the studies that assessed the renal safety of TDF in Africa used eGFR only as renal function assessment tool. Limited studies in Africa assessed tubular dysfunction associated with TDF using serum phosphate measurement and urine dipstick for proteinuria and glycosuria [25]. Besides, studies showed the absence of APOL1 genetic variants in people of Ethiopian ancestry and the less susceptibility Ethiopians to HIVAN [26, 27] which may suggest a relatively better renal function at initiation of ART. However, there are no data on the effect of ART on renal function in this population. Therefore, we aimed to assess factors associated with renal function during the first year on ART with special focus on TDF in Ethiopian HIV positive adults.

## Method

### Study setting and participants

HIV positive adults who were eligible for ART at Jimma University Specialized Hospital, Jimma Health Centre and Agaro Health Centre were invited to participate in a nutritional supplementation trial with initiation of ART from July 2010 to August 2012 [28]. ART eligibility criteria were i) CD4 count < 200 cells/mm^3^, ii) CD4 count <350cells/mm^3^ and WHO stage 3 or 4 disease, or iii) WHO stage 4 disease regardless of CD4 count. ART was provided for HIV patients for free. The available first-line nucleoside reverse transcriptase inhibitors (NRTI) were TDF, zidovudine (AZT), stavudine (d4T), lamivudine (3TC) and abacavir and the available non-nucleoside reverse transcriptase inhibitors (NNRTI) were efavirenz (EFV) and nevirapine (NVP). The preferred first line antiretroviral regimens in Ethiopia guideline during the study period were TDF/3TC/EFV, AZT/3TC/NVP or AZT/3TC/EFV [24]. HIV positive persons who were ≥ 18 years of age, not pregnant or lactating, BMI > 16 kg/m^2^ with no current use of nutritional supplements and living within 50 km of the recruitment sites were invited to participate in the trial [28]. An additional 30 HIV patients who were excluded from the nutritional trial due to a BMI < 16 kg/m^2^ were included in the present study.

### Demographic and clinical data

Demographic data were collected by trained study nurses who used structured questionnaires in the local languages Amharic or Afaan Oromo. Clinical data were collected by health professionals working in the ART clinics at baseline and after 3, 6 and 12 months. Blood pressure was measured using blood pressure monitor after minimum of 5 min of rest; two readings 1 min apart were recorded and the average was taken.

### Anthropometry and body composition

Weight and height were measured at baseline and after 6 and 12 months with calibrated scales and stadiometers, respectively, with the participant barefoot and wearing minimal clothing and body mass index (BMI) was calculated as weight (kg)/(height (m))^2^. Body composition (fat free mass (FFM) and fat mass (FM)) in HIV patients was assessed using the deuterium dilution method at the baseline and after 6 months [29]. Fat free mass index (FFMI) was calculated as [FFM (kg)/height (m)^2^] and fat mass index (FMI) was calculated as [FM (kg)/height (m)^2^] [30].

### Laboratory

The study laboratory personnel collected 10 ml of fasting venous blood in an EDTA tube, and 10 ml in a plain tube. CD4+ T cells were enumerated using the Facscount® (Becton-Dickinson, New Jersey, USA). Spot urine samples were also collected, and urine dipstick analyses were performed to check for proteinuria and glycosuria at baseline, 3, 6, and 12 months.

Creatinine was measured at baseline, 3, 6 and 12 months using a colorimetric kinetic assay (HORIBA ABX A11A01933) for Pentra 400 (HORIBA ABX, Montpellier, France). The glomerular filtration rate was estimated using the “Chronic Kidney Disease Epidemiology Collaboration” (CKD-EPI) [31] equation without ethnic correction as our published data [32] and previous studies showed that CKD-EPI equation without ethnic correction provides the best estimate of GFR in an African population [33, 34]. Highly sensitive C-reactive protein (CRP) was measured at baseline using a latex enhanced immunoturbidimetric assay (HORIBA ABX A11A01611) for Pentra 400 (HORIBA ABC, Montpellier, France). Serum concentration of inorganic phosphate (mmol/L) was measured using the automated Humastar 80 analyzer (Human Diagnostics, Wiesbaden, Germany). The test (Human diagnostics ref. no 10027) was based on a modified ammonium molybdate principle. The accuracy was monitored using commercial control samples (Humatrol N, 13511 and Humatrol P, 13512).

HIV-1 RNA load was quantified using a commercial PCR assay (RealTime HIV-1, Abbott Laboratories, Illinois, USA) at baseline and at 6 months using an automated extraction system (m2000 Real Time System, Abbott Laboratories, Illinois, USA). Immunochromatographic rapid test was used for qualitative detection of HCV antibody and Hepatitis B surface antigen (HBsAg) in serum at baseline (SD Bioline, Korea).

### Statistical analysis

Data were described with frequencies, medians (interquartile ranges [IQR]) and mean (±Standard deviation (SD)). Demographic and clinical characteristics were compared between patients initiated on TDF and non-TDF based ART regimen using Wilcoxon rank-sum test or Pearson chi square test, as appropriate. Age, sex, hypertension, WHO stage, tuberculosis treatment, HCV, HBsAg and viral load suppression at 6 months were used as categorical variable. All anthropometric, body composition and other laboratory data (CRP, CD4 count, serum phosphate, viral load at baseline and eGFR) were used as continuous variables. Univariate and multivariate linear regression analysis were used to determine factors associated with baseline eGFR. Sociodemographic, anthropometric, body composition and clinical parameters were included in univariate linear regression analysis and variables that were associated with eGFR with a *P*-value < 0.1 were included in multivariate linear regression analysis. Linear mixed model was used to evaluate 12-month eGFR difference in TDF and non-TDF based regimen. Univariate and multivariate linear regression models were used to assess how baseline characteristics and changes in viral load and body composition after 6 months of intervention (ART and nutritional supplementation) affected 12-month eGFR; only variables with a *P*-value < 0.1 in the univariate analysis were included in multivariate analysis. We also further analyzed by categorizing change in eGFR (12 month –baseline) in to < − 3, − 3 to 3, > 3 and < − 10, − 10 to 10 and > 10 and assessed factors associated with each group eGFR change with univariate logistic regression. Stata version 14.0 (StataCorp, Texas, USA) was used for all analyses.

## Results

**Fig. 1:**
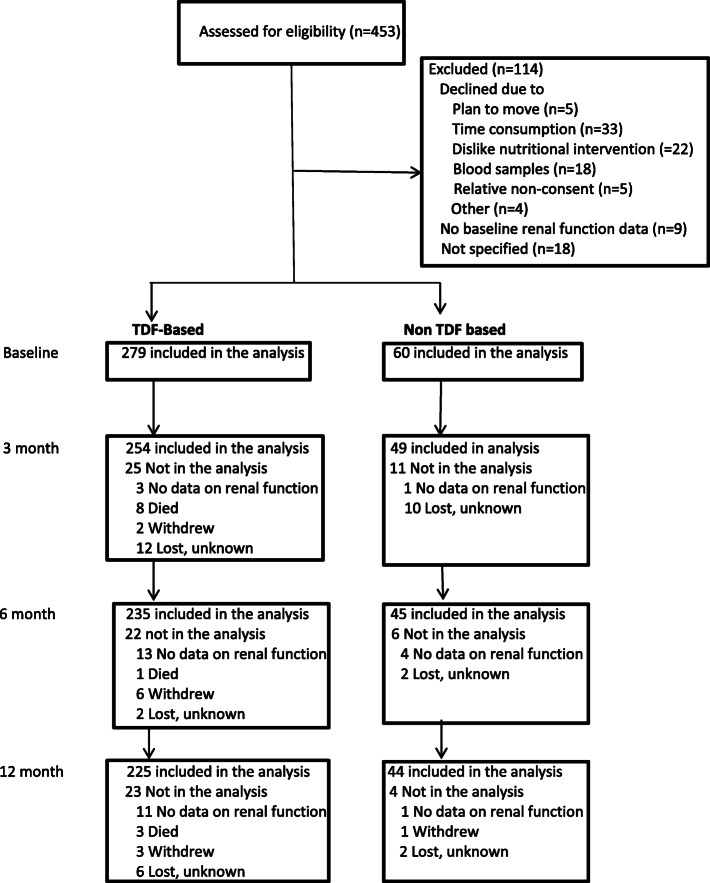
Flow chart of enrolment of participants and follow up based on treatment assignment (Tenofovir (TDF) and non TDF based). Note that participants with no renal function data on one visit had renal functon data on the subsequent visits except one participant in non-TDF based group who did not have renal function data both at 6th and 12th month

A total of 340 HIV positive adults had baseline renal function tests performed and 339 were initiated on ART. Of these, 279 (82.3%) were initiated on a TDF based regimen (Fig. 1). More specifically; 239 (70.5%) were on TDF/3TC/EFV, 40 (11.8%) were on TDF/3TC/NVP, 4 (1.2%) were on AZT/3TC/EFV, 49 (14.4%) were on AZT/3TC/NVP, 1 (0.3%) was on d4T/3TC/EFV and 6 (1.8%) were on d4T/3TC/NVP. More HIV positive adults on TDF based regimen were males, older, had higher fat-free mass and were taller than HIV positive adults started on non-TDF based regimen.

None of the HIV positive adults had known history of diabetes mellitus and chronic kidney disease, 0.9% (*n* = 3) of the HIV positive adults reported history of hypertension and 2.6% (*n* = 9) reported history of cigarette smoking. Only 1.8% (*n* = 6) had hypertension (systolic blood pressure > 140 mmHg or diastolic blood pressure > 90 mmHg). Two of these participants had both systolic and diastolic hypertension and four of them had only diastolic hypertension. All the six participants were on TDF based regimen. Hepatitis C virus antibody was positive in 0.6% (*n* = 2) of participants and both participants were on TDF based regimens.

There was no difference in baseline eGFR in HIV positive adults initiated on TDF and non-TDF based regimen (Table 1).

**Table 1:** Baseline characteristics of 340 HIV positive adults by antiretroviral treatment initiated^1^

	Tenofovir based regimen (*n* = 279)	Non tenofovir-based regimen (*n* = 60)	*P*
Age, years			< 0.001
18–25	50 (17.9)	22 (36.7)	
26–35	134 (48.0)	31 (51.7)	
36–45	66 (23.7)	6 (10.0)	
> 45	29 (10.4)	1 (1.7)	
Sex			0.003
Males	101 (36.2)	10 (16.7)	
Females	178 (63.8)	50 (83.3)	
WHO Stage			0.78
I	84 (30.1)	15 (25.0)	
II	82 (29.4)	19 (31.7)	
III	89 (31.9)	22 (36.7)	
IV	24 (8.6)	4 (6.7)	
Weight, kg	49.2 (44.1; 54.3)	47.3 (42.1; 51.3)	0.08
Fat Free mass, Kg	39.0 (35.2; 44.8)	37.1 (33.7; 40.1)	0.01
Fat mass, kg	8.4 (6.0; 11.9)	9.9 (7.2; 11.6)	0.49
Height, m	1.60 (1.54; 1.67)	1.6 (1.5; 1.6)	0.02
Body mass index, kg/m^2^	18.9 (17.4; 20.6)	18.3 (17.2; 20.5)	0.78
On tuberculosis treatment, Yes	30 (10.8)	5 (8.3)	0.58
C-reactive protein, mg/L	2.0 (0.6, 7.7)	1.9 (0.4; 6.8)	0.57
CD4 count, cells/ul	178 (111; 243)	192 (122; 231)	0.45
Serum phosphate, mmol/L^a^	1.3 (0.3))	1.3 (0.3))	0.89
Hepatitis B surface antigen positive^2^, Yes	10 (3.7)	2 (3.6)	0.97
Viral load, log (copies+ 1/mL)	4.8 (4.3; 5.4)	4.8 (4.3; 5.2)	0.79
Urine Protein dipstick positive^3^, Yes	14 (2.3)	1 (6.6)	0.28
eGFR, ml/min/1.73m^2 a^	117.7 (21.7)	122 (18.0)	0.13

*eGFR* Estimated glomerular filtration rate.

^1^ Data shown as median (IQR) or n (%) and ^a^ mean (±SD)

^2^*n* = 330 ^3^*n* = 255

In univariate linear regression analysis, lower baseline eGFR was significantly associated with older age, male gender, higher CRP, higher weight and presence of hypertension. Older age and high CRP level remained after adjustment for other variables in multivariate analysis (Table 2). Being a female was a strong protective factor in univariate analysis but shifted to be a risk factor in multivariate analysis with a borderline significant for lower eGFR. Adjusting the analysis for age changed the relation as males were significantly older than females (*P* < 0.05). A large proportion of females (*n* = 181, 79.2%) were younger than 35 years whereas only 50.9% (*n* = 57) of males were younger than 35 years. However, when formally testing, no interaction was found between age and sex (*P* = 0.2).

**Table 2:** Factors associated with estimated glomerular filtration rate at baseline in univariate and multivariate linear regression in 340 antiretroviral naïve HIV positive adults in Ethiopia. Values are coefficient B, 95% confidence interval (CI) and *P* value

	Univariate		Multivariate	
	B (95%CI)	*P*	B (95%CI)	*P*
Age, years				< 0.001
18–25	–			
26–35	−11.1 (−16.2; − 5.89)	< 0.001	−11.6 (−17.0; −6.3)	< 0.001
36–45	−24.0 (−30.1; − 17.92)	< 0.001	−25.1(−31.6; − 18.7)	< 0.001
> 45	−33.9 (− 41.8; − 25.93)	< 0.001	−35.5 (− 43.9; − 27.1)	< 0.001
Sex, Female	5.1 (0.3; 9.8)	0.04	−4.6 (− 9.6; 0.4)	0.07
WHO Stage				
I	–	–	–	–
II	0.1 (−5.8; 6.0)	0.98	–	–
III	1.4 (−4.3; 7.2)	0.62	–	–
IV	− 1.7 (− 10.7; 7.1)	0.69	–	–
Weight, kg	−0.3 (− 0.5; 0.01)	0.06	− 0.2 (− 0.5; 0.1)	0.17
Fat Free mass, Kg	− 0.2 (− 0.5; 0.2)	0.32	–	–
Fat mass, kg	− 0.3 (− 0.8; 0.1)	0.15	–	–
Height, m	− 0.2 (− 0.5; 0.06)	0.13	–	–
Body mass index, kg/m^2^	− 0.4 (− 1.2; 0.5)	0.42	–	–
Fat mass index, kg/m^2^	− 0.5 (− 1.6; 0.6)	0.34	–	–
Fat free mass index, kg/m^2^	− 0.03 (−1.4; 1.4)	0.96	–	–
Hypertension, Yes	−15.7 (−32.8; 1.3)	0.07	−6.6 (−22.0; 8.7)	0.39
C-reactive protein, mg/L	−0.1 (− 0.2; 0.01)	0.08	− 0.1 (− 0.2; − 0.02)	0.02
CD4 count, cells/ul	−0.002 (− 0.02; 0.02)	0.88	–	–
On tuberculosis treatment, Yes	6.1 (−1.3; 13.5)	0.11	–	–
Hepatitis B surface antigen positive^1^, Yes	1.8 (−10.0; 13.7)	0.76	–	–
Hepatitis C virus antibody Positive^2^, Yes	18.8 (−10.8; 48.3)	0.21	–	–
Viral load, log (copies+ 1/mL)	0.8 (−1.4; 3.2)	0.45	–	–

^1^*n* = 330, ^2^*n* = 318

Renal function data were available for 79% (n=269) of HIV positive adults at 12 months after ART initiation; 80% (n=225) for patients in TDF regimen and 73% (n=44) for patients in non-TDF based regimen (Fig. 1). Antiretroviral drug substitution was made for 21 patients in the first 12 months of ART. Nineteen of the substitutions were affecting the choice of NNRTI; either from NVP to EFV (*n* = 9) or from EFV to NVP (*n* = 10) due to toxicity, new tuberculosis treatment or pregnancy. Two patients had NRTI substitutions; one from d4T to AZT and the other from AZT to TDF due to toxicity. Patient whose ART changed from AZT to TDF at third month was excluded from follow up analysis comparing TDF based vs non-TDF based ART. However, no patients were changed from TDF to other NRTI’s.

**Fig. 2:**
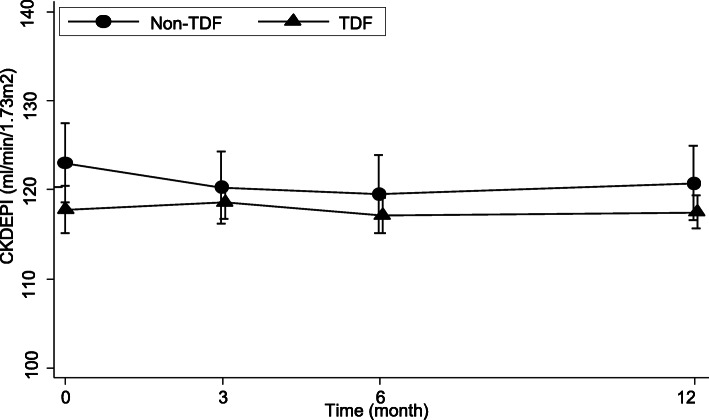
Mean with 95% Confidence interval estimated glomerular filtration rate (eGFR) using creatinine based Chronic Kidney Disease Epidemiology Collaboration eGFR equation during 12 months of ART with and without tenofovir (TDF) based regimen

The median (IQR) and mean (± SD) change in eGFR with 12 months of ART was 0.8 (− 11.1; 10.0) ml/min/1.73m^2^ and − 0.3 (18.8) ml/min/1.73m^2^ respectively. None of the HIV positive adults had decline to < 60 ml/min/1.73m^2^. From four HIV positive adults that had eGFR < 60 ml/min/1.73m^2^ at the baseline, three of them had eGFR of > 90 ml/min/1.73m^2^ at 12 months and one had lost follow up after the baseline. There was no difference in change of creatinine and eGFR from baseline to 12 months between patients starting TDF based regimen and non-TDF based regimen (Table 3, Fig. 2). Serum phosphate levels were higher in HIV positive adults on TDF based regimen for 12 months (Table 3). Urine dipstick was performed for 257 patients at baseline and 106 of patients had urine dipstick results at all four time points; at baseline, 3, 6 and 12 months. Proteinuria was detected in 5.8% (15/ 257), 3.9% (8/204), 3.2% (7/220) and 2% (4/196) of patients at baseline, 3, 6 and 12 months, respectively. All urine dipstick positive results were + 1 and + 2 (which is < 100 mg/dl) except one patient at baseline who had + 3 (500 mg/dl) and lost follow up. There was no difference in proportion of proteinuria between the four time points (*P* = 0.19). Except in one patient at baseline and 1 patient at 12 month, all reported proteinuria was in participants taking TDF based regimen. However, except the four participants with proteinuria on urine dipstick at 12 month, all participants with proteinuria had at least two urine dipstick results and none of the participants had proteinuria in two consecutive urine samples. Glycosuria was detected in 2 patients on TDF based regimen at 3 months but was not persistent and in 2 other patients on non-TDF based regimen at 12 months.

**Table 3:** Change in renal function parameters through 12 months based on antiretroviral treatment regimen initiated. Values are mean (± SD) and *P* value

	Tenofovir based regimen *n* = 225	Non tenofovir based regimen *n* = 43	*P*
Serum creatinine, mg/dl	− 0.01 (0.2)	−0.007 (0.1)	0.86
Serum phosphate^a^, mmol/L	0.5 (0.9)	0.1 (1.2)	0.04
eGFR, ml/min/1.73m^2^	−0.4 (−19.6)	− 0.2 (14.8)	0.95

^a^*n* = 182 for tenofovir group and 40 for non tenofovir group

Older HIV positive adults and those with unsuppressed viral load at 6 month of ART had lower eGFR at 12 months of ART initiation. Moreover, patients with a higher baseline eGFR had also higher eGFR at 12 months (Table 4). However, we found no difference in 12 month eGFR between HIV patients initiated on TDF based regimen and non-TDF based regimen.

**Table 4:** Factors associated with 12 month estimated glomerular filtration rates in HIV positive adults in Ethiopia. Values are from univariate and multivariate linear regression with coefficient B, 95% confidence interval (CI) and *P* value

	Univariate		Multivariate	
	B (95%CI)	*P*	B (95%CI)	*P*
Age, y				
18–25	–			
26–35	−8.4 (− 12.4; − 4.5)	< 0.001	−5.5 (− 9.5; − 1.4)	0.008^+^
36–45	− 14.6 (− 19.3; − 9.9)	< 0.001	− 9.7 (− 14.6; − 4.7)	< 0.001^+^
> 45	−22.6 (− 28.7; − 16.5)	< 0.001	− 15.4 (− 22.1; − 8.7)	< 0.001^+^
Sex, Female	1.7 (− 1.9; 5.2)	0.36	–	–
**Baseline characteristics**				
Body mass index, kg/m^2^	−0.2 (− 0.9; 0.4)	0.66	–	–
Fat mass index, kg/m^2^	−0.3 (− 1.2; 0.5)	0.46		–
Fat free mass index, kg/m^2^	−0.2 (− 1.2; 0.8)	0.72	–	–
Hypertension, Yes	0.01 (− 6; 6)	0.99	–	–
C-reactive protein, mg/L	−0.01 (− 0.1; 0.07)	0.99	–	–
CD4 count, cells/ul	0.002 (−0.01; 0.02)	0.83	–	–
On tuberculosis treatment, Yes	−1.8 (− 7.5; 3.9)	0.53	–	–
Hepatitis B surface antigen positive, Yes	4.2 (− 5.3; 13.8)	0.38	–	–
Hepatitis C virus antibody Positive, Yes	0.5 (−26.2; 27.3)	0.97	–	–
Viral load, log (copies+ 1/mL)	0.3 (−1.4; 2.1)	0.68	–	–
eGFR, ml/min/1.73 m2	0.3 (0.23; 0.38)	< 0.001	0.2 (0.1; 0.3)	< 0.001^+^
**Six month change**				
Change in body mass index, kg/m^2^	0.1 (−1; 1.2)	0.87	–	–
Change in fat mass index, kg/m^2^	−0.4 (−1.8; 1)	0.58	–	–
Change in fat free mass index, kg/m^2^	0.8 (−1; 2.6)	0.37	–	–
Change in CD4 count, cells/ul	0.03 (0.01; 0.05)	< 0.001	0.01 (−0.005; 0.02)	0.18^+^
Viral load > 1000 copies/ml at 6 months, Yes	−15.1 (−23.4; −6.8)	< 0.001	−9.9 (− 17.2; − 2.5)	0.009^+^
**Antiretroviral treatment**				
TDF Treatment	−2.5 (−7; 2)	0.28	0.2 (−3.8; 4.2)	0.93*

^+^ Variables in one model

* Value are after adjustment for all variables that were included in multivariate model

Based on previous studies which described an association between a drop in eGFR > 3 mL/min/1.73 m^2^ per year and increased mortality and morbidity [35, 36], we performed further analysis by categorizing the participants based on 12 months change in eGFR into three groups: “decliner” if drop in eGFR > 3 mL/min/1.73 m^2^, “stable” if change in eGFR remained between − 3 and 3 mL/min/1.73 m^2^ and “riser” if there were an increase in eGFR of > 3 mL/min/1.73 m^2^. Of 268 patients who had 12 months renal function data, 41, 14.6 and 44.4% were “decliner”, “stable” and “riser”, respectively. About 40.9, 14.7, and 44.4% in TDF group and 41.9, 14, and 44.1% in non-TDF group were “decliner”, “stable” and “riser” respectively. We found no difference in baseline characteristics (age, sex, weight, BMI, FM, FFM, WHO clinical staging, CRP, HBV and HCV antibody positivity, CD4 count and viral load level) between these three groups (*P* > 0.05). In univariate logistic regression, there was no significant associations between “decliners” or “risers” compared with “stable” eGFR; “decliner” compared with “riser”; and “decliner” compared with the combined group of “stable” and “riser” and any baseline characteristics, 6 month changes and type of ART regimen (TDF and non-TDF based). The only exception was baseline eGFR for all comparisons. HIV positive adults with higher baseline eGFR were more likely to be “decliner” than “stable” or “riser” and were more likely to be “stable” compared to “riser”. We also found similar results when using cut-offs of − 10 and 10 ml/min/1.73 m^2^, and there were no significant differences between TDF and non-TDF based regimen (*P* > 0.05). About 26.9, 48.5 and 24.6% in the whole participants with ART; 28, 45.8, and 26.2% in TDF group; and 20.9, 62.8, and 16.3% in non-TDF group had eGFR drop greater than 10 mL/min/1.73 m^2^, change between − 10 to 10 mL/min/1.73 m^2^ and increase greater than > 10 mL/min/1.73 m^2^ at 12 month from the baseline eGFR, respectively**.**

We further compared the baseline characteristics of HIV positive persons who completed 12 months follow up and had eGFR data with those who did not have 12 month eGFR data. We did not find any difference in age, sex and other baseline characteristics like fat mass and fat free mass, CD4 count, Viral load, presence of HBV and TB infection and baseline eGFR. However, HIV positive persons that had no data at 12 months had higher baseline CRP, lower weight and BMI at baseline and more patients were in WHO Stage III than stage I compared to patients who completed follow up and had 12 month data.

## Discussion

As ART is a lifelong treatment, it is important to understand the potential adverse effects of treatment among various populations. We studied HIV treatment effect on the renal function determining eGFR, serum phosphate and urine dipstick test in HIV patients during the first year on ART. We did not find any significant negative effect on renal function associated with neither TDF use nor other antiretroviral drugs. We found that older individuals and HIV positive adults with high CRP had lower eGFR at ART initiation. Moreover, older and HIV positive adults with unsuppressed viral load at 6 months experienced declines in eGFR after 12 months of ART.

Most HIV positive adults were initiated on fixed dose combination drugs either TDF/3TC/EFV, which was recommended as preferred first line regimen in all treatment naïve HIV patients without contraindication, or AZT/3TC/NVP, which was recommended in women of child bearing age without usage of reliable contraceptive measures [24]. This is the likely reason why HIV positive adults in the non-TDF group were younger and more were females compared with patients in the TDF group.

Most studies have shown that lower CD4 count and advanced HIV disease are associated with lower eGFR in ART naïve HIV patients [2, 37]. However, our study did not find this which may be because the vast majority of our study population had pronounced immunosuppression and/or advanced HIV disease, thus making comparison with patients with higher CD4 counts difficult.

We found that a higher CRP was associated with lower eGFR in ART naïve HIV positive adults. However, in a previous paper we showed that CRP was not associated with serum creatinine [32]. The association of CRP with eGFR but not with serum creatinine may be because serum creatinine production is affected by other factors like age and muscle mass which are considered in eGFR equations to assess renal function [38]. AIDS Clinical Trials Group study A5224 showed the inverse correlation of systemic inflammatory markers and eGFR in HIV patients both before and during ART using the equation CKD-EPI with cystatin C-creatinine (CysC-Cr), suggesting that systemic inflammation may result in renal impairment [39]. However, no association was found between CRP and eGFR when eGFR was determined by CKD-EPI Cr equation in AIDS Clinical Trials Group study [39]. A previous study has shown that the performance of CKD-EPI CysC-Cr equation in HIV patients was better compared to using cystatin C or creatinine alone [40]. Therefore, the different results in the association of CRP and eGFR may be related to the use of appropriate markers for eGFR equations for the population.

ART has been reported to improve renal function [41–45]; and most studies indicated that the renal function improvement was mostly observed in HIV patients with baseline renal impairment [42–44]. We also found that 44.4 and 24.6% had an increase in eGFR greater than 3 and 10 mL/min/1.73m^2^ at 12 month, respectively and none of the patients had decline to < 60 ml/min/1.73m^2^. However, we did not find general difference in eGFR in HIV patients before ART and after 12 months of ART. This may be because most of the HIV positive adults in our cohort had normal renal function at the initiation of ART. The absence of APOL1 genetic variants in people of Ethiopian ancestry which makes Ethiopians less susceptibility to HIVAN may be the contributing factor for relatively normal kidney function at initiation of ART [26, 27]. It may also be related to the performance of the eGFR equation which is not validated for the population, although we found that the CKDEPI better estimated creatinine clearance compared to other eGFR equations in this population [32]. We found that HIV positive adults with higher baseline had a decrease in eGFR from baseline. However, these patients still had a higher eGFR at 12 month which was seen in linear regression model. The fact that HIV positive adults with higher baseline eGFR were more likely to be decliners compared to stable or riser may indicate statistically regression to the mean eGFR for high values which was also seen by the instability in the median and IQR of change in eGFR.

Our data also showed no difference in serum creatinine and eGFR in patients who were on TDF based and non-TDF based regimen after 12 months of ART. Moreover, no persistent proteinuria or glycosuria as markers of tubular dysfunction was observed in the study population. Additionally, as opposed to TDF nephrotoxicity, the serum phosphate level was higher in TDF based regimen compared to non-TDF based regimen after 12 months of ART. Although larger retrospective cohort studies reported decline in kidney function with TDF use in Africa [21–23], there are also studies that showed no significant association of TDF use with renal impairment [41, 46, 47]. The different results and reports on renal safety of TDF in various populations may suggest that TDF associated kidney injury may be developing in genetically predisposed populations. However, as TDF accumulates in the renal cortex after administration [48], the nephrotoxic effect may also be a cumulative effect and may be observed with longer time exposure to TDF as reported in other studies for antiretroviral drugs [10, 16]. Additionally, it may also be due to the small sample size and participants characteristics of our cohort that we did not find the association of TDF use with renal impairment. In contrast to most studies which confirmed the association of TDF with renal impairment [16, 49], our cohort of HIV population were younger, had normal kidney function at the time of ART initiation and had less concomitant traditional risk factors and were not taking other antiretroviral drugs such as atazanavir and lopinavir boosted with ritonavir which have been shown to increase the risk of renal impairment when co-administered with TDF [50].

These results on TDF/non-TDF and eGFR remained in adjusted linear regression model including all variables associated in univariate linear regression model (Table 4). However, older patients had lower eGFR before ART initiation and a further decline in eGFR was observed in these groups with ART. This association has also been reported previously as generally eGFR decreases with increasing age [10]. Our data also showed that patients who had high viral load at 6 months after ART initiation had a lower eGFR at 12 month. Though we had no data on CRP at 6 month, these patients may have systemic inflammation which may have resulted in reduction in eGFR.

Our study monitored the renal function of a cohort of HIV patients that were followed at regular interval with repeated measurements of serum creatinine and urine dipstick during the first 12 months of ART. Though the sex and age distribution of our cohort of HIV patients well represents the HIV prevalence in Ethiopia and the epidemiology in sub-Saharan Africa, we had limited HIV patients with concomitant traditional risk factors for kidney disease and also the hepatitis B and C co-infection rate was lower than previous report in Ethiopia [51] . Also data on concomitant medications other than for opportunistic disease were not collected on the participants, preventing us from making any conclusion in these groups. As the study was also primarily designed for nutritional intervention trial, the nutritional intervention may also have an effect on serum creatinine metabolism and can have an impact the eGFR change. In addition, few patients were in non-TDF based ART regimen at 12 months and this reduced the power of the study and increased the risk of type II error.

## Conclusion

We found no significant difference in kidney function in HIV positive adults treated with TDF or non-TDF NNRTI based ART regimen for 12 months. However, older HIV positive persons especially those above 45 years and those with unsuppressed viral load while on ART seem at higher risk of developing renal impairment and deserve special focus on renal monitoring. Moreover, data in larger cohort on longer term safety and slowly accumulating adverse events of TDF (> 1 year) are still warranted in this population.

## Supplementary information

**Additional file 1.**

## Data Availability

All data generated or analyzed during this study are included in this published article. Data on the treatment and baseline and 12 month estimated glomerular filtration rate is included on Additional file 1.

## Abbreviations

3TC: Lamivudine
ART: Antiretroviral treatment
AZT: Zidovudine
BMI: Body mass index
CKD-EPI: Chronic kidney disease epidemiology collaboration
CRP: C-reactive protein
CysC-Cr: Cystatin C-creatinine
d4T: Stavudine
EFV: Efavirenz
eGFR: Estimated glomerular filtration rate
FFM: Fat free mass
FFMI: Fat free mass index
FM: Fat mass
FMI: Fat mass index
HBsAg: Hepatitis B surface antigen
HCV: Hepatitis C virus
HIVAN: HIV associated nephropathies
IQR: Interquartile range
NNRTI: Non-nucleoside reverse transcriptase inhibitor
NRTI: Nucleoside reverse transcriptase inhibitor
NVP: Nevirapine
SD: Standard deviation
TDF: Tenofovir disoproxil fumarate
WHO: World health organization
